# Impact of tumor motion on target delineation and dose calculation accuracy using rapid‐acquisition HyperSight CBCT in online adaptive radiotherapy

**DOI:** 10.1002/acm2.70428

**Published:** 2025-12-28

**Authors:** Yi‐Fang Wang, Fan Liu, Michael J. Price, Adam C. Riegel

**Affiliations:** ^1^ Department of Radiation Oncology Vagelos College of Physicians Surgeons Columbia University Irving Medical Center New York New York USA

**Keywords:** CBCT, motion management, online adaptive radiotherapy

## Abstract

**Background:**

HyperSight CBCT offers rapid image acquisition and enhanced image quality, supporting its integration into online adaptive radiotherapy (OART). However, its performance under respiratory‐induced tumor motion requires further evaluation.

**Purpose:**

This study evaluates the feasibility of using rapid‐acquisition HyperSight CBCT for internal target volume (ITV) delineation and dose calculation in the setting of respiratory motion, with implications for OART workflows.

**Methods:**

A dynamic thoracic phantom simulating superior‐inferior tumor motion (5–25 mm amplitude from the motion center, 6‐second cycle time) was imaged using Varian's HyperSight CBCT on the Ethos platform. Four reconstruction algorithms were evaluated: filtered back projection (FDK), iterative CBCT (iCBCT), iCBCT with Acuros, and metal artifact reduction (MAR). Experiments included variation of cycle times (4, 6, and 8 s), breathing patterns (Cos^6^, sinusoidal, and hysteresis), and repeat acquisitions to assess the impact of scan initiation time relative to the respiratory cycle. In addition to fast scans, slow acquisitions (60‐second cycle) were performed for further evaluation. Reference datasets included 4DCT maximum intensity projection (MIP) and average intensity projection (AIP) for ITV delineation and HU comparison. Volumetric modulated arc therapy plans were created on AIP images and recalculated on HyperSight images. Dose distributions were compared using gamma analysis (1%/1 mm, 10% threshold).

**Results:**

For the Cos^6^ breathing pattern with a 6‐second cycle time, HyperSight CBCT produced ITV volumes and HU values comparable to 4DCT at small respiratory amplitudes (≤10 mm), with Dice similarity coefficients exceeding 0.8 and dose calculations passing 1%/1 mm gamma analysis. At larger amplitudes (≥15 mm), ITVs were underestimated to 28–40% of the reference values, with Dice coefficients falling below 0.45 and increasing image distortion. Longer cycle times (8s) and irregular breathing patterns (hysteresis) further reduced trajectory visualization, while more uniform motion (sinusoidal) improved trajectory coverage but still showed image deformation at 15mm amplitude. Repeat acquisitions demonstrated varying image representations at different scan initiation times, particularly with the longer 8‐second cycle. Slow‐scan CBCT achieved close agreement with 4DCT MIP and AIP in ITV volumes and HU values.

**Conclusions:**

HyperSight CBCT is feasible for OART in patients with limited respiratory motion, regular breathing pattern and short breathing cycle time. For larger excursions, slow scans or motion management strategies may be required to ensure accurate target delineation and dose calculation.

## 1 INTRODUCTION

Cone‐beam computed tomography (CBCT) has been a cornerstone imaging modality in image‐guided radiotherapy (IGRT) for over a decade, enabling patient setup verification and alignment with sub‐millimeter precision.^1^ Despite its widespread use, the image quality of CBCT remains inferior to that of fan‐beam CT, which is traditionally used for treatment planning and dose calculation.^2^ A recent advancement in CBCT technology, the HyperSight system (Varian Medical Systems, Palo Alto, CA), has significantly improved CBCT image quality, facilitating its use in online adaptive radiotherapy.^3^

The enhanced capabilities of HyperSight CBCT extend beyond patient setup and alignment; its superior image quality now supports tasks such as contouring, treatment planning, and dose calculation while the patient remains on the treatment couch. These advancements are mainly due to innovations in detector technology and enhanced reconstruction algorithms.^4^ HyperSight CBCT employs detectors with active areas twice that of conventional CBCT systems and utilizes a Cesium Iodide (CsI) based scintillator. This configuration ensures rapid readout, minimal image lag, and improved x‐ray conversion efficiency.^4^ Additionally, the system enables rapid image acquisition, completing a scan in approximately 6 s using a full‐fan, half‐arc trajectory—faster than conventional CBCT acquisitions, which typically require about 33 s for a full‐fan, half‐arc scan or up to 60 s for a standard half‐fan, full‐arc protocol.^5^ Beyond hardware enhancements, HyperSight CBCT incorporates advanced image reconstruction algorithms, including iterative CBCT (iCBCT), scatter‐corrected iCBCT using Acuros computed tomography scatter (CTS) correction (iCBCT Acuros), and metal artifact reduction (MAR). The MAR algorithm minimizes artifacts caused by high‐density materials such as metal implants, ensuring accurate and reliable imaging in complex clinical scenarios.^4^ These features collectively contribute to producing high‐quality images suitable for adaptive radiotherapy workflows.

In online adaptive radiotherapy, where accurate re‐contouring of the target volume is essential for minimizing target margins, the visualization of tumor motion trajectories plays a crucial role. This capability enhances adaptive radiotherapy by ensuring robust target coverage while optimizing dose sparing for organs at risk (OARs). The ability to account for tumor motion can be particularly beneficial for treatment sites characterized by large and variable tumor motion.^3^

Although numerous studies have highlighted the significant image quality improvements achieved with HyperSight CBCT,^5^, ^6^, ^7^, ^8^, ^9^, ^10^, ^11^, ^12^ limited research has investigated the impact of short acquisition time on tumor motion management, internal target volume (ITV) delineation, motion trajectory visualization, and changes in Hounsfield Unit (HU) values for dose calculation. In conventional radiotherapy, fan‐beam 4DCT scanning combined with maximum intensity projection (MIP) is routinely employed at the CT simulation stage to capture the full range of tumor motion and define the ITV, while free‐breathing scans or average intensity projection (AIP) are used for dose calculation.^13^ Ideally, HyperSight CBCT should capture the complete tumor motion trajectory when used for online replanning in adaptive radiotherapy, particularly when respiratory gating or breath‐hold techniques are not employed. Additionally, HyperSight CBCT should provide reliable HU values for dose calculation when utilized in online dose calculation workflows. However, the rapid acquisition time of HyperSight CBCT presents a unique challenge: While it effectively reduces motion artifacts, it may limit the ability to characterize the full range of tumor motion.

Recent studies have begun to explore the application of HyperSight CBCT under respiratory motion using motion phantoms. Koo et al. investigated IGRT shifts between ITVs derived from 4DCT and HyperSight CBCT, reporting good positional consistency for regular breathing motions with a shift difference below 5 mm.^14^ Zhao et al. analyzed geometric shifts of ITV centroids between HyperSight and 4DCT AIP images, demonstrating high alignment accuracy for small amplitudes (< 5 mm) and short breathing cycles (< 6 s), with minor differences in ITV shape.^5^ Oliver et al. evaluated motion artifacts and HU variation in HyperSight scans using a QUASAR respiratory phantom with moderate (1.5–5.5 mm) peak‐to‐center motion amplitudes, finding minimal dosimetric impact but visible motion artifacts.^15^

This study investigates the impact of HyperSight's rapid acquisition time on internal target volume (ITV) determination, tumor motion trajectory visualization, and the reliability of HU values for online dose calculation. Specifically, it evaluates the visualization of tumor trajectories and HU variation across clinically applicable motion amplitudes of 5, 10, 15, 20 mm, with an additional 25 mm amplitude case included to examine system performance under extreme motion conditions.^13^ These scenarios were analyzed using four reconstruction algorithms: Filtered Back Projection (FDK), iCBCT, iCBCT Acuros, and MAR. To further characterize the influence of breathing dynamics, different respiratory cycle times (4, 6, and 8 s) and patterns (sinusoidal, Cos,^6^ and hysteresis) were investigated, and 10 repeated scans were performed serially to assess the impact of different scan starting points. To establish a robust comparison, the HyperSight CBCT images were benchmarked against 4DCT data sets, which served as the ground truth. Additionally, the performance of HyperSight's slow CBCT was investigated as an alternative when rapid acquisitions fail to fully capture tumor motion. The findings of this study are particularly significant for online adaptive radiotherapy, especially for treatment sites that exhibit significant respiratory motion, such as the lung, liver, and upper abdominal tumors. By comprehensively analyzing tumor motion and HU reliability across varying conditions, this study provides insights into the feasibility of using HyperSight CBCT for real‐time planning and dose calculation.

## 2 MATERIAL AND METHODS

### 2.1 Phantom and image data acquisition

#### 2.1.1 Phantom

A CIRS dynamic thorax phantom containing a 2 cm‐diameter spherical tumor target was employed to simulate respiratory‐induced tumor motion (CIRS, Inc., Norfolk, VA, USA). The phantom dimensions closely resemble those of an average adult human thorax. Tumor motion was simulated by translating a lung‐equivalent rod embedded with the tissue‐equivalent target within the phantom.

#### 2.1.2 Data acquisition

All scans were performed on the Ethos platform (version 2.0, Varian Medical Systems) using HyperSight CBCT with 125 kVp and 261 mAs. CBCT images were reconstructed with four algorithms: FDK, iCBCT, iCBCT Acuros, and MAR. The voxel size of all HyperSight images was 0.105 × 0.105 × 0.2 cm^3^. For reference, each motion scenario was scanned on a radiotherapy CT simulator (SOMATOM Definition AS, Siemens Healthineers) at 120 kVp, 0.5 s rotation, and 88 mAs, reconstructed with a voxel size of 0.097 × 0.097 × 0.2 cm^3^. Amplitude‐based 4DCT reconstruction was performed using the AZ‐733V respiratory gating system (Anzai Medical Inc.) for external motion tracking. MIP images generated from the 4DCT were used as reference datasets for internal target volume (ITV) evaluation, and AIP images were used for HU and dosimetric comparison.

#### 2.1.3 Simulated breathing patterns

A Cos^6^ waveform was used to simulate an idealized human breathing pattern with superior‐inferior (SI) displacement amplitudes of 5, 10, 15, 20, and 25 mm. In this study, amplitude is defined as the maximum displacement from the motion center; thus, the corresponding peak‐to‐peak motion ranges were 10, 20, 30, 40, and 50 mm, respectively. The waveform was selected as each cycle features a broad valley, indicating a relatively prolonged exhalation phase, and a short, sharp peak that signifies the inhalation phase. A 6‐second cycle time was applied for all amplitudes, except for the 25 mm displacements, which required a 7.6‐second cycle due to mechanical motion velocity constraints. In addition to these baseline acquisitions, further experiments were conducted to evaluate the influence of respiratory cycle and pattern variations. Different respiratory cycle times (4, 6, and 8 s) were simulated using the Cos^6^ waveform with iCBCT Acuros reconstruction at three motion amplitudes (5, 10, and 15 mm). To investigate the impact of different breathing patterns, sinusoidal and Cos^6^ waveforms with superior–inferior amplitudes of 5, 10, and 15 mm were simulated using 6‐ and 8‐second cycles, along with a hysteresis pattern incorporating anterior–posterior (2.5 mm), lateral (1 mm), and superior–inferior (5, 10, and 15 mm) displacements, all reconstructed with iCBCT Acuros. Finally, to assess the impact of scan starting point, 10 repeated acquisitions were performed using a 6‐second Cos^6^ waveform at 10 and 20 mm amplitudes, and an 8‐second cycle for both the Cos^6^ and sinusoidal waveforms at a 20 mm amplitude. In practice, it is difficult to precisely control the initiation of scanning at a specific respiratory phase because of inherent preparation and imaging delays. By acquiring 10 sequential scans while the target was in continuous motion, different starting points within the breathing cycle were effectively sampled, allowing evaluation of how scan initiation timing influences motion depiction. In addition to HyperSight fast scans, slow acquisition scans with a cycle time of approximately 60 s were performed using the Cos^6^ pattern and iCBCT Acuros reconstruction algorithm for motion amplitudes of 5, 10, and 20 mm to assess the impact of extended acquisition time on motion representation and image quality. Appendix A shows all measured scans corresponding to each section of the results.

### 2.2 Image data processing

All acquired CBCT and CT images were imported into the Eclipse treatment planning system (version 15.6, Varian Medical System, Palo Alto, CA) for ITV segmentation and image registration. The reference ITVs were generated from the MIP images of the conventional 4DCT scans. For segmentation, an initial contour was generated using a threshold of −600 HU, followed by manual editing to refine the ITV shape detected and exclude artifacts. This approach was designed to reflect the clinical workflow, in which physicians adjust the ITV volumes at the treatment console. To ensure consistency, the Eclipse lung window and level settings (window level: −375 HU, window width: 625 HU) were applied during contouring for all images. Three experienced, board‐certified medical physicists were involved in editing and reviewing the contours, and a consensus contour was finalized to ensure accuracy and reliability.

To compare the volume and the HU values of each ITV for all investigated imaging modalities and motion amplitudes against the reference ITV, rigid registration between the Hypersight images and the reference CT was performed using Eclipse. Registration alignment was evaluated based on the outer shape of the solid phantom rather than the moving spherical target, as the phantom geometry remained consistent across all acquisitions. Consequently, registration was straightforward, and its accuracy was verified through visual inspection. The ITV structure from the reference CT (ITV‐CT) was mapped onto the Hypersight images to facilitate the comparison.

Characterizing ITV contours is critical for this study. Three regions were delineated on the imaging dataset: (1) ITV delineated on the Hypersight images (ITV‐Hyper), (2) ITV‐CT from reference 4DCT MIP, and (3) the region within ITV‐CT but not in ITV‐Hyper (non‐ITV). Although visually inspected by experts, a statistical analysis using *Z*‐tests was performed as an additional confirmation—specifically to determine whether the mean HU of the ITV‐Hyper region was significantly different from that of the surrounding non‐ITV region. A significance level of *α* < 0.05 was used to determine statistical significance.

### 2.3 Quantitative comparison of ITV volumes and dice similarity coefficients

For comparison, the ITV volumes derived from each CBCT reconstruction algorithm were quantitatively analyzed against the corresponding ITV volumes from the 4DCT MIP for each amplitude of motion. The analysis included the calculation of the ratio of the ITV volume (ITV‐Hyper) from each CBCT to the ITV volume (ITV‐CT) of the reference CT, as well as the Dice similarity coefficient between the ITV‐Hyper and ITV‐CT volumes.

### 2.4 HU value comparison

The mean and standard deviation of HU values within the ITV derived from Hypersight images were compared to the corresponding values from the reference CTs (MIP and AIP) across the examined breathing motion. These HU differences and variations were used to assess the consistency and robustness of image quality, as HU distribution stability indirectly influences the accuracy of electron density assignment and, consequently, the reliability of target delineation and dose calculation on HyperSight images.

### 2.5 Dose calculation comparison

Corresponding dose distributions were then evaluated on both HyperSight and reference image sets. Volumetric modulated arc therapy (VMAT) plans were initially created on the 4DCT AIP images using two arcs with 6 MV photon energy and the Acuros dose calculation algorithm, prescribing a uniform dose to the ITV plus an additional 5 mm isotropic margin. These plans were subsequently recalculated on the HyperSight images with the iCBCT Acuros reconstruction algorithm, which, along with MAR, is supported by the Ethos system for direct dose calculation on CBCT. This comparison was designed to isolate the dose differences arising purely from the HU difference. Dose distribution accuracy was assessed using gamma analysis with 1%/1 mm criteria and a 10% dose threshold. Only cases with 5 mm and 10 mm motion amplitudes were included in the analysis, as the ITVs delineated on HyperSight closely matched those from the reference 4DCT. For larger motion amplitudes (15–25 mm), the HyperSight ITVs volumes were substantially different from the reference ITVs, making direct plan recalculation unrealistic clinically.

## 3 RESULTS

### 3.1 Baseline analysis: ITV volume and geometric comparison across motion amplitudes and reconstruction algorithms

**FIGURE 1:**
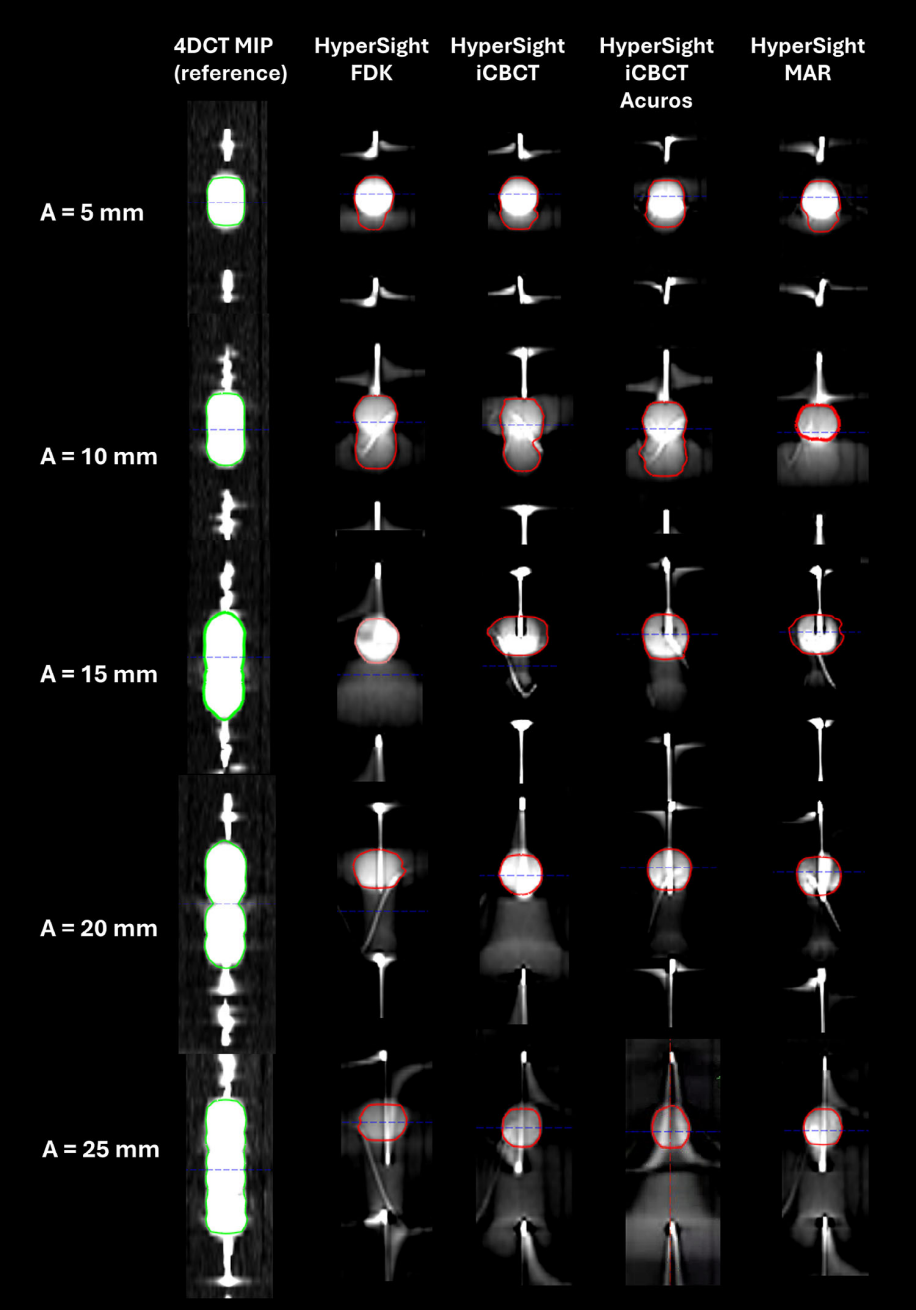
Coronal views of the motion phantom images acquired using HyperSight and 4DCT MIP across the tested superior‐inferior motion amplitudes. A, amplitude; MAR, metal artifact reduction; MIP, maximum intensity projection.

Coronal views of the motion phantom images are presented in Figure 1. ITV contoured on HyperSight CBCT images—reconstructed with four algorithms (FDK, iCBCT, iCBCT Acuros, and MAR)—were compared to 4DCT MIP images, which served as the reference standard for each SI amplitude. At a 5 mm amplitude, all four HyperSight algorithms produced ITVs (FDK: 6.2 cc, iCBCT: 6.7 cc, iCBCT Acuros: 6.8 cc, MAR: 6.2 cc) that closely matched the reference (6.6 cc), with the most significant deviation being 0.4 cc. Similarly, at a 10 mm amplitude, three of the HyperSight reconstructions (FDK: 9.7 cc, iCBCT: 9.3 cc, iCBCT Acuros: 10 cc) yielded ITVs comparable to the reference (9.7 cc), whereas MAR generated a notably smaller volume (5 cc). Dice similarity coefficients (DSCs) for both 5 mm and 10 mm motion amplitudes exceeded 0.8 across all reconstruction methods, with the exception of the MAR algorithm.

**FIGURE 2:**
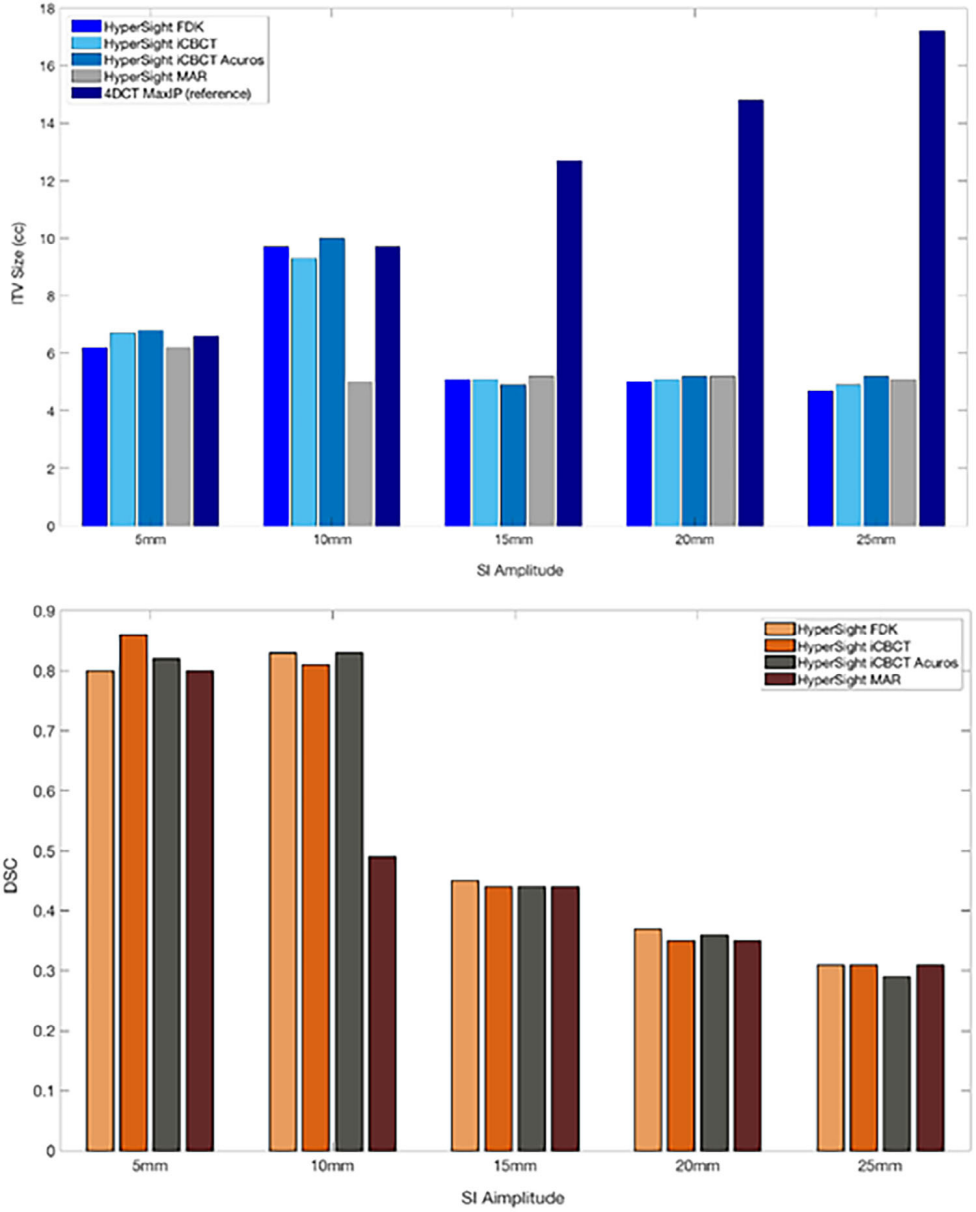
Bar plots of internal target volume (ITV) measurements and Dice similarity coefficient (DSC) obtained from HyperSight CBCT and 4DCT MIP across the tested superior‐inferior (SI) motion amplitudes. ITV, internal target volume; MAR, metal artifact reduction; MIP, maximum intensity projection; and SI, superior‐inferior.

For amplitudes of 15 mm or greater, the ITVs derived from HyperSight reconstructions deviated substantially from the reference. At 15 mm amplitude, the volumes from FDK, iCBCT, iCBCT Acuros, and MAR (5.1 cc, 5.1 cc, 4.9 cc, and 5.2 cc, respectively) were approximately 40% of the reference volume (12.7 cc). These discrepancies increased for the 20 mm amplitude analysis (FDK: 5 cc, iCBCT: 5.1 cc, iCBCT Acuros: 5.2 cc, MAR: 5.2 cc), reaching only 34% of the reference (14.8 cc). At the less frequent 25 mm amplitude, the HyperSight‐defined volumes (FDK: 4.7 cc, iCBCT: 4.9 cc, iCBCT Acuros: 5.1 cc, MAR: 5.1 cc) were only 28% of the reference (17.2 cc). DSC also declined with increasing motion amplitude across all reconstruction methods, with values of approximately 0.44, 0.35, and 0.31 at 15, 20, and 25 mm amplitudes, respectively. A bar plot comparing the ITV sizes and DSC values derived from each HyperSight reconstruction and the reference scans is presented in Figure 2, with corresponding quantitative data provided in Table 1. These findings indicate a considerable risk of target underdosing if ITVs are delineated solely on HyperSight rapid‐acquisition images for relatively larger‐amplitude respiratory motion.

**TABLE 1:** Internal target volume (ITV) measurements obtained from HyperSight CBCT using various reconstruction algorithms, compared with reference ITV values derived from 4DCT maximum intensity projection (MIP) images.

	ITV Volume (cc)					Dice Similarity Coefficient (DSC) to the Reference ITV			
Amplitude	CT MIP (reference)	HyperSight FDK	HyperSight iCBCT	HyperSightiCBCT Acuros	HyperSight MAR	HyperSightFDK	HyperSight iCBCT	HyperSightiCBCT Acuros	HyperSight MAR
5 mm	6.6	6.2	6.7	6.8	6.2	0.80	0.86	0.82	0.80
10 mm	9.6	9.6	9.3	9.9	5	0.83	0.81	0.83	0.49
15 mm	12.7	5.1	5.1	4.9	5.2	0.45	0.44	0.44	0.44
20 mm	14.8	5.0	5.1	5.2	5.2	0.37	0.35	0.36	0.35
25 mm	17.2	4.7	4.9	5.1	5.1	0.31	0.31	0.29	0.31

Dice similarity coefficients (DSC) between HyperSight‐ and MIP‐based ITVs are included to quantify volumetric agreement.

### 3.2 Baseline analysis: Image intensity evaluation

**FIGURE 3:**
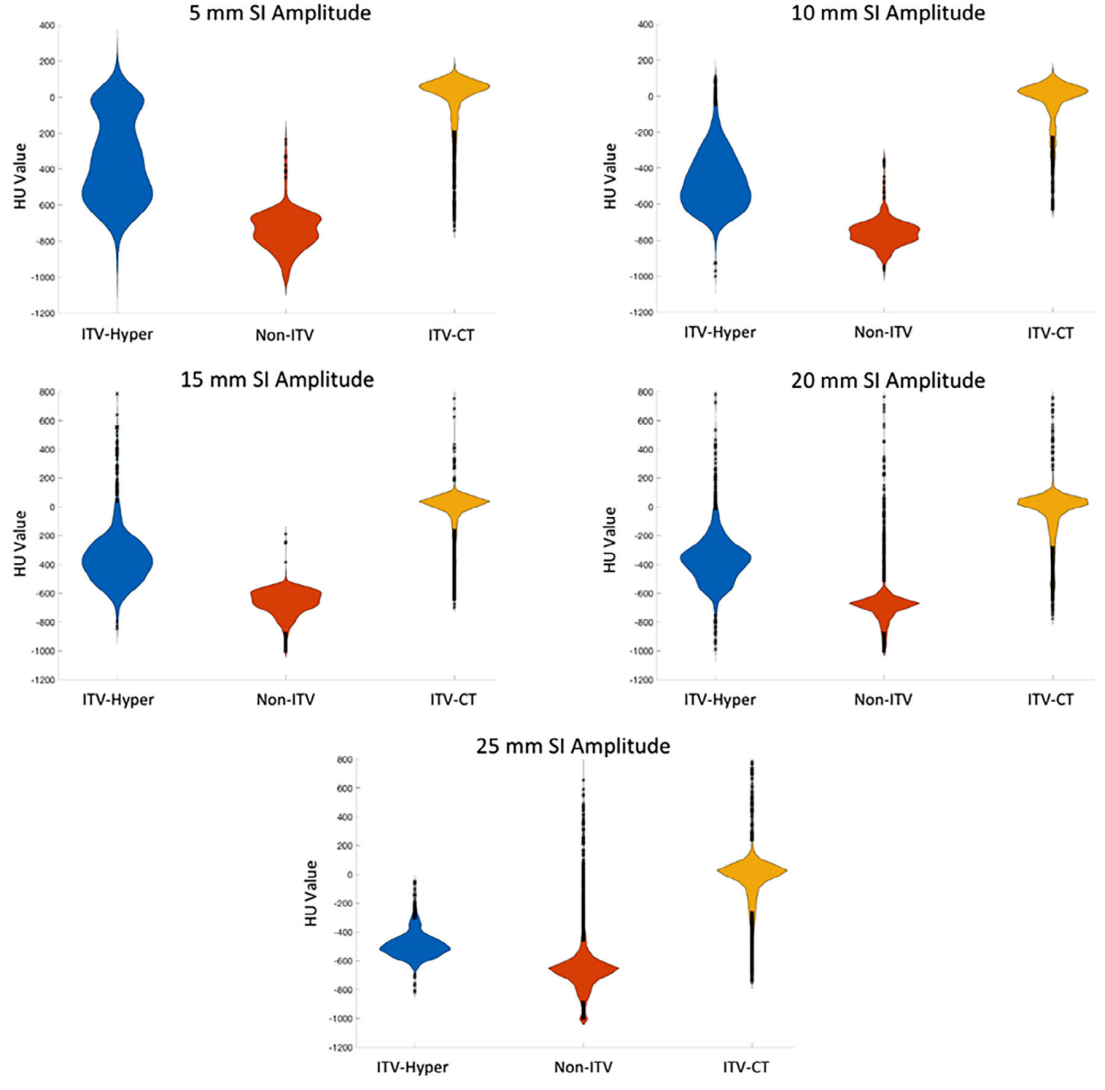
Violin plots of HU values within the internal target volume on HyperSight CBCT (ITV‐Hyper), the non‐ITV region on HyperSight CBCT, and the ITV on 4DCT MIP (ITV‐CT) across the examined superior‐inferior (SI) motion amplitudes. The HyperSight data presented are based on images reconstructed with the iCBCT Acuros algorithm. ITV, internal target volume, and SI, superior‐inferior.

In addition to ITV volumes, HU values within the respective ITVs contoured on each HyperSight dataset were compared with 4DCT MIP images to assess ITV visualization and with 4DCT AIP images to evaluate dose calculation accuracy. Figure 3 presents violin plots comparing HU distributions across all imaging modalities and motion amplitudes. Detailed numerical data are provided in Table 2. From the violin plots, HyperSight CBCT shows a broader HU distribution within the ITV compared to 4DCT MIP. This difference is expected, as MIP is a post‐processed reconstruction that combines all respiratory phases using the highest‐intensity pixels, while HyperSight CBCT represents a single rapid scan. The broader HU spectrum observed with HyperSight CBCT highlights the greater contouring and dose calculation uncertainty that may arise in online imaging. Across all amplitudes, the mean HU values within the ITV on HyperSight CBCT decreased with increasing motion amplitude, making the target less distinct for segmentation. Non‐ITV regions—defined as areas included in the 4DCT‐derived ITV but missed by HyperSight CBCT—showed substantially lower HU values than the corresponding HyperSight ITV regions. In all cases, mean HU values within the ITV were around 150 HU higher than in non‐ITV regions, with statistically significant differences (*p* <  0.0002), supporting that ITV underestimation arose from imaging limitations rather than contouring bias.

**TABLE 2:** Mean Hounsfield Unit (HU) values ± standard deviation within the internal target volume (ITV) from HyperSight CBCT, 4DCT maximum intensity projection (MIP), and average intensity projection (AIP) images.

	CT Numbers within ITV (Mean HU ± SD)						CT Numbers within non‐ITV (Mean HU ± SD)			
Amplitude	CT AIP (reference for dose calculation)	CT MIP (reference for contouring)	HyperSight FDK	HyperSight iCBCT	HyperSight iCBCT Acuros	HyperSight MAR	HyperSight FDK	HyperSight iCBCT	HyperSight iCBCT Acuros	HyperSight MAR
5 mm	−352.1 ± 221.4	−18.9 ± 130.1	−323.6 ± 221.4	−351.2 ± 227.3	−342.0 ± 228.0	−323.9 ± 223.2	−707.2 ± 110.7	−750.5 ± 97	−747.1 ± 78.7	−686.9 ± 130.4
10 mm	−473.6 ± 173.9	−26.8 ± 125.9	−485.8 ± 140.5	−481.4 ± 140.6	−473.5 ± 147.7	−465.2 ± 153.8	−759.8 ± 70.5	−709.8 ± 141.0	−774 ± 47.4	−728.4 ± 93.2
15 mm	−530.9 ± 192.6	−10.4 ± 184.1	−353.6 ± 148.1	−421.2 ± 169.8	−358.8 ± 170.3	−386.9 ± 182.9	−693.3 ± 77.4	−653.1 ± 99.7	−658.5 ± 80.5	−652.0 ± 91.8
20 mm	−580.9 ± 151	−22.4 ± 235.5	−380.4 ± 141.2	−414.8 ± 135.7	−364.9 ± 166.9	−431.1 ± 160.0	−696.5 ± 146.2	−677.5 ± 180.7	−684.1 ± 175.1	−660.3 ± 124.9
25 mm	−624.6 ± 119.4	−15.5 ± 225.3	−500.1 ± 69.5	−477.9 ± 84.9	−495.6 ± 74.9	−467.8 ± 94.5	−672.1 ± 145.6	−687.5 ± 170.8	−649.9 ± 138.4	−666.8 ± 171.9

HU values within non‐ITV regions from HyperSight CBCT images are also included.

To evaluate the accuracy of dose calculation, HU values from HyperSight images were compared with those from 4DCT AIP images, which represent the clinically relevant reference for dose calculation accuracy. At motion amplitudes of 5 and 10 mm, the mean (± standard deviation) ITV HU values on 4DCT AIP were −352.1 ± 221.4 and −473.6 ± 173.9, respectively, closely matching those from HyperSight CBCT with maximum differences of approximately 20 HU. This indicates that HyperSight CBCT provides HU accuracy suitable for dose calculation in limited‐motion scenarios. However, for larger motion amplitudes (> 10 mm), the ITV contours on HyperSight images fail to capture the full motion trajectory and thus do not match those on AIP images, limiting the validity of HU comparison.

### 3.3 Effect of breathing cycle time

To investigate the impact of respiratory motion characteristics on image acquisition, HyperSight CBCT was evaluated using varying cycle durations, breathing waveforms, and scan start time relative to the respiratory phase. As summarized in Table 3, ITV volumes at 4, 6, and 8‐second cycles were largely comparable and consistent with those from 4DCT MIP at 5 and 10 mm amplitude. At 15 mm amplitude, however, the reconstructed ITV volume was lower than the MIP‐defined ITV, indicating incomplete representation of the motion envelope. Analysis of voxel intensities showed that mean HU values within the HyperSight‐defined ITV were generally consistent with those measured on AIP images, with the exception of the 15 mm amplitude case. The findings in Section 3.5 further indicate that, for the 8‐second cycle time, image appearance and ITV position can vary depending on the scan start timing.

**TABLE 3:** Comparison of ITV volumes and mean HU values obtained from HyperSight CBCT and 4DCT reconstructions under varying respiratory motion conditions.

	ITV volume (cc)			CT numbers within ITV (Mean HU ± SD)			
Amplitude	5 mm	10mm	15mm	Amplitude	5 mm	10mm	15mm
HyperSight t=4s	6.5	9.5	5.0	HyperSight t=4s	−318.7 ± 235.6	−462.0 ± 170.2	−397.3 ± 149.6
HyperSight t=6s	6.8	9.9	4.9	HyperSight t=6s	−342.0 ± 228.0	−473.5 ± 147.7	−358.8 ± 170.3
HyperSight t=8s	6.6	9.6	5.0	HyperSight t=8s	−345.3 ± 225.2	−448.0 ± 157.8	−446.8 ± 158.7
4DCT MIP *t* = 4s	6.5	9.7	12.7	4DCT AIP *t* = 4s	−333.5 ± 237.5	−455.7 ± 171.9	−535.6 ± 163.3
4DCT MIP *t* = 6s	6.6	9.6	12.7	4DCT AIP *t* = 6s	−352.1 ± 221.4	−473.6 ± 173.9	−530.9 ± 192.6
4DCT MIP *t* = 8s	6.7	9.7	12.7	4DCT AIP *t* = 8s	−336.7 ± 235.2	−468.8 ± 178.6	−539.7 ± 109.7

Simulations were performed with cycle times of 4, 6, and 8 s and motion amplitudes of 5, 10, and 15 mm using a Cos^6^ breathing waveform. ITV volumes were delineated on HyperSight images and compared with reference 4DCT MIP, while mean HU values within the ITV were compared with those from 4DCT AIP.

### 3.4 Effect of breathing pattern

**FIGURE 4:**
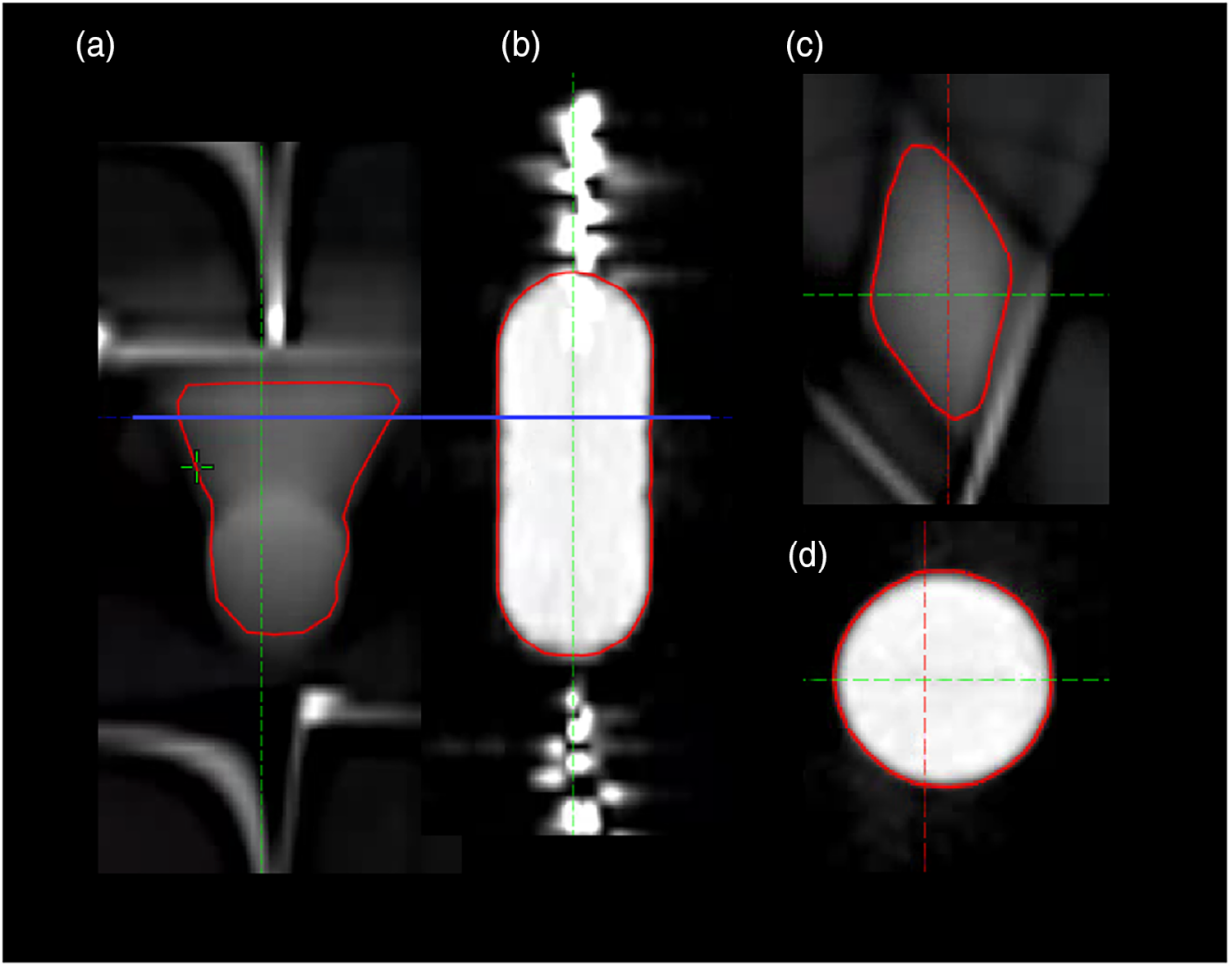
Comparison of target visualization for sinusoidal motion with 15 mm amplitude. (a) Sagittal view from HyperSight CBCT with contoured ITV. (b) Sagittal view from 4DCT MIP reconstruction. (c) Axial slice from HyperSight, and (d) axial slice from 4DCT MIP, both corresponding to the location indicated by the blue line in (a) and (b).

The results for different respiratory motion patterns are summarized in Table 4. For the sinusoidal pattern, a substantial reduction in ITV volume relative to 4DCT MIP was observed at 15 mm amplitude. The measured ITV volume of 10.3 cc was larger than that obtained with the Cos^6^ pattern (5.2 cc), reflecting the more uniform motion distribution in sinusoidal breathing. However, although part of the motion trajectory was sampled, the reconstructed target exhibited pronounced geometric deformation due to angular undersampling in the presence of motion (Figure 4), complicating accurate contour delineation. A similar effect was observed for hysteresis at 15 mm amplitude. For hysteresis, irregular target motion resulted in incomplete trajectory sampling even at 10 mm amplitude, yielding an ITV volume approximately 1 cc smaller than that measured on 4DCT MIP. With respect to intensity analysis, mean HU values within the HyperSight‐defined ITV for sinusoidal and hysteresis patterns were generally consistent with those from 4DCT AIP. We also evaluated the effect of cycle time on sinusoidal and hysteresis patterns. Similar to the Cos^6^ pattern, ITV volumes and mean HU values were consistent between 6‐ and 8‐second cycle times. For the sinusoidal pattern, differences were 0.3 cc/6 HU at 10 mm and 0.5 cc/25 HU at 15 mm amplitudes; for hysteresis, 0.3 cc/8 HU at 10 mm and 0.6 cc/22 HU at 15 mm. It should be noted that, as shown in Section 3.5, for the 8‐second cycle time, the results can vary depending on the scan start timing.

**TABLE 4:** Comparison of ITV volumes and mean HU values obtained from HyperSight CBCT and 4DCT reconstructions under varying respiratory motion conditions.

	ITV volume (cc)			CT numbers within ITV (Mean HU ± SD)			
Amplitude	5 mm	10mm	15mm	Amplitude	5 mm	10mm	15mm
HyperSight Cos6	6.8	9.9	4.9	HyperSight Cos6	−342.0 ± 228.0	−473.5 ± 147.7	−358.8 ± 170.3
HyperSight Sin	7.6	10.3	10.3	HyperSight Sin	−364.8 ± 234.7	−458.9 ± 140.3	−506.1 ± 87.1
HyperSight Hysteresis	7.3	9.5	12.6	HyperSight Hysteresis	−348.9± 223.9	−448.4 ± 117.8	−527.4 ± 134.4
4DCT MIP Cos6	6.6	9.6	12.7	4DCT AIP Cos6	−352.1 ± 221.4	−473.6 ± 173.9	−530.9 ± 192.6
4DCT MIP Sin	7.4	10.5	13.3	4DCT AIP Sin	−326.5 ± 226.2	−455.2 ± 140.4	−522.6 ± 98.7
4DCT MIP Hysteresis	7.7	10.5	15.4	4DCT AIP Hysteresis	−352.1± 216.2	−463.2 ± 136.9	−552.0 ± 105.0

Simulations were performed with different motion patterns, Cos,^6^ sinusoidal, and hysteresis, and motion amplitudes of 5, 10, and 15 mm using a 6‐second cycle time. ITV volumes were delineated on HyperSight images and compared with reference 4DCT MIP, while mean HU values within the ITV were compared with those from 4DCT AIP.

### 3.5 Effect of scan start timing

**FIGURE 5:**
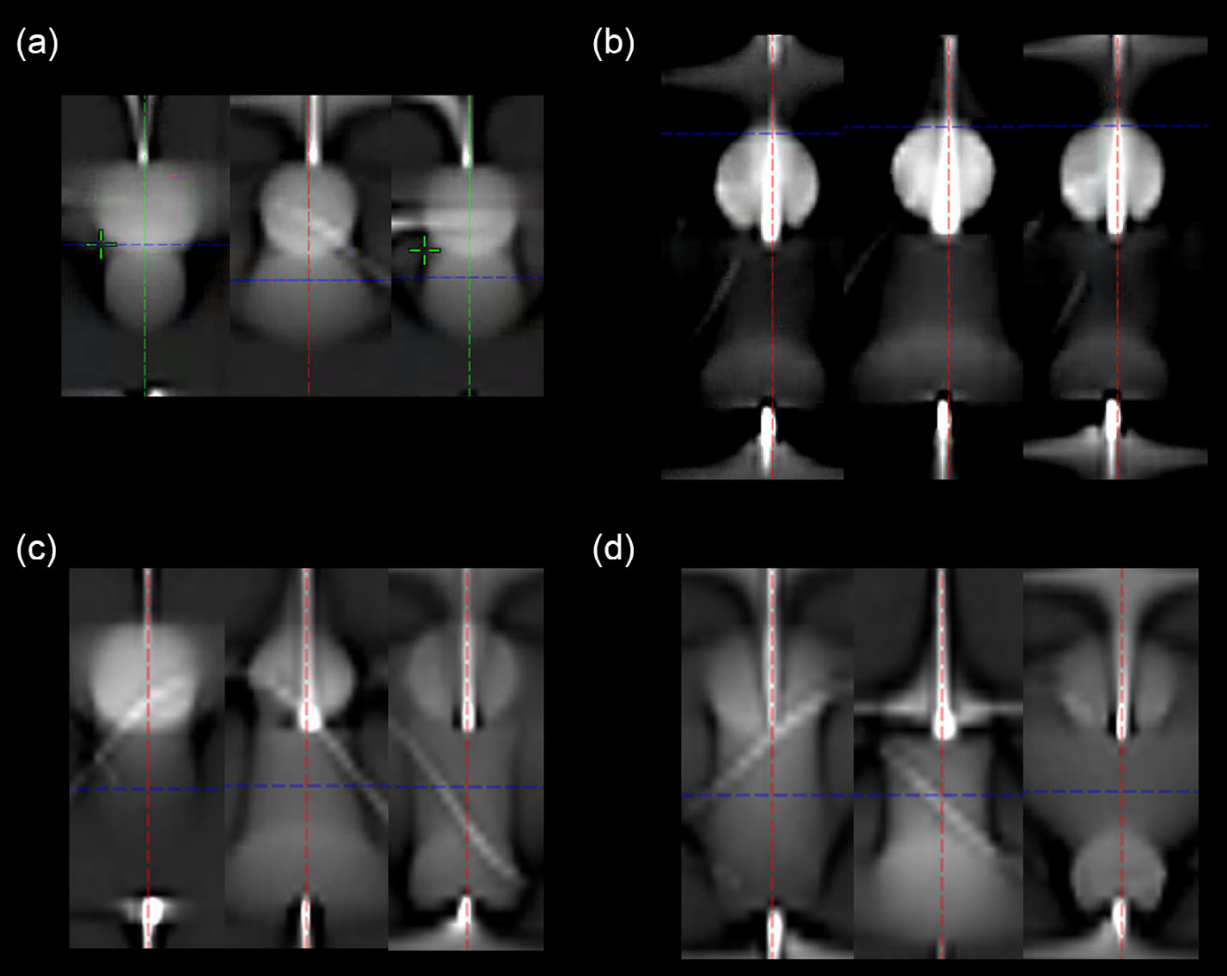
Comparison of target visualization across repeated scans: (a) 10 mm amplitude, 6‐second cycle, Cos^6^ waveform; (b) 20 mm amplitude, 6‐second cycle, Cos^6^ waveform; (c) 20 mm amplitude, 8‐second cycle, Cos^6^ waveform; and (d) 20 mm amplitude, 8‐second cycle, sinusoidal waveform.

Precise control of scan initiation is difficult, so 10 sequential scans were acquired while the target was in continuous motion to sample different respiratory phases and assess their impact on motion depiction. For the 6‐second Cos^6^ waveform at 10 mm amplitude, all 10 scans yielded consistent ITV volumes and mean HU values. The average ITV volume was 9.4 cc, with a maximum inter‐scan difference of 0.2 cc, and the mean HU was 480, with a maximum difference of 8 HU. For the 6‐second Cos^6^ waveform at 20 mm amplitude (Figure 5a and b), similar results were observed, with a maximum ITV volume difference of 0.1 cc and a mean HU variation of approximately 44 HU, though missing motion trajectories were consistently present across scans.

To further evaluate the effect of scan onset timing under slower motion, 10 repeated scans were performed using an 8‐second cycle with both the Cos^6^ and sinusoidal waveforms at a 20 mm amplitude. As shown in Figure 5c and d, image appearance began to change more noticeably across repeated scans, reflecting the influence of varying acquisition start phases. For the 8‐second Cos^6^ waveform, the maximum ITV volume difference reached 2.9 cc, with a corresponding HU variation of 242 HU. For the sinusoidal waveform, the maximum ITV volume difference was 3.2 cc, and the HU variation was 27 HU. The contoured ITVs could be located at different positions within the breathing cycle, depending on the scan start time.

### 3.6 Evaluation of slow CBCT acquisition

The ITV volume and CT number obtained from HyperSight slow CBCT were compared to reference values derived from 4DCT MIP for volume and AIP for CT number. As shown in Table 5, the ITV volumes and HU values from the slow CBCT scans closely matched those from the 4DCT references, with volume differences within 1 cc and CT number within 15 HU.

**TABLE 5:** Internal target volume (ITV) measurements derived from 4DCT maximum intensity projection (MIP) and HyperSight slow CBCT, along with mean Hounsfield Unit (HU) values ± standard deviation within the ITV from 4DCT average intensity projection (AIP) and HyperSight slow CBCT.

	ITV volume (cc)			CT numbers within ITV (Mean HU ± SD)		
Amplitude	CT MIP (reference for contouring)	HyperSight Slow CBCT	HyperSight 6‐second CBCT	CT AIP (reference for dose calculation)	HyperSight Slow CBCT	HyperSight 6‐second CBCT
5 mm	6.6	7	6.8	−352.1 ± 221.4	−347.5 ± 243.8	−342.0 ± 228
10 mm	9.6	9.5	9.9	−473.6 ± 173.9	−488.2 ± 178.9	−473.5 ± 147.7
20 mm	14.8	14.0	5.2	−580.9 ± 151	−594.0 ± 166.6	−364.9 ± 166.9

### 3.7 Dose distribution comparison

**FIGURE 6:**
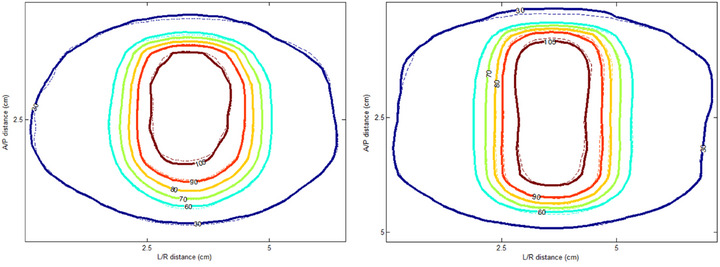
Comparison of isodose lines on the sagittal plane between dose calculations based on HyperSight Acuros images (dashed lines) and 4DCT AIP images (solid lines) for 5 mm (left) and 10 mm (right) motion amplitudes. A/P, anterior/posterior; L/R, left/right.

Lastly, dose distributions were calculated on both 4DCT AIP and HyperSight iCBCT Acuros datasets and evaluated using gamma analysis with 1%/1 mm criteria and a 10% dose threshold. For the Cos^6^ pattern, 5 mm motion amplitude and 6 s cycle time, the gamma passing rates were 98.1%, 96.0%, and 95.3% in the axial, sagittal, and coronal planes through the ITV center, respectively, with a 3D passing rate of 95.6%. For the 10 mm amplitude, the corresponding passing rates were 99.8%, 95.9%, and 95.2%, with a 3D passing rate of 94.8%. Figure 6 shows the isodose lines comparison. These results indicate that HyperSight images enable dose calculations with accuracy comparable to 4DCT AIP, supporting their potential utility in online adaptive radiotherapy for patients with limited respiratory motion.

## 4 DISCUSSION

Previous studies have examined ITV estimation and registration accuracy between conventional CBCT and 4DCT‐derived images, showing good alignment but larger MIP‐derived ITVs and small registration errors in the millimeter range.^16^, ^17^, ^18^ Conventional CBCT typically requires about 30 s to 1 min for image acquisition, covering the full respiratory range, whereas the newly developed HyperSight CBCT achieves rapid 6‐second acquisition, which may not fully visualize the entire motion trajectory. As CBCT evolves from a tool for patient positioning to a dataset for segmentation and dose calculation in online adaptive radiotherapy, this study aims to establish benchmark data characterizing the performance and limitations of HyperSight CBCT under controlled phantom conditions with defined motion amplitudes, breathing cycles, and motion patterns. The findings offer valuable insight into the potential of HyperSight CBCT to support adaptive radiotherapy workflows.

Our results demonstrate that HyperSight CBCT yields ITV sizes comparable to 4DCT MIP at small motion amplitudes (≤ 10 mm) using Cos^6^ breathing pattern and 6‐second cycle time, suggesting its potential utility in cases with limited respiratory excursion. For both 5 mm and 10 mm amplitudes, deviations in ITV volumes were minimal (≤0.4 cc) compared to 4DCT MIP images, and HU values within the ITV were consistent with those from 4DCT Average images. A discrepancy in image appearance was observed with the MAR algorithm, which yielded a smaller ITV size for the 10 mm amplitude. This may be due to the reconstruction algorithm slightly smoothing the target's motion trajectory by treating it as an artifact during image processing. Moreover, dose calculations performed on HyperSight iCBCT Acuros images passed the stringent 1%/1 mm gamma criteria with > 95% agreement across all planes, supporting the clinical feasibility of using HyperSight CBCT for dose calculation in patients with limited motion.

However, significant discrepancies emerged as the motion amplitude increased beyond 10 mm. At amplitudes of 15 mm and above, ITVs derived from HyperSight CBCT represented only 28%–40% of the corresponding 4DCT MIP volumes, with Dice similarity coefficients ranging from 0.29 to 0.45 across all reconstruction algorithms. This underestimation reflects the inability of fast‐acquisition CBCT to visualize the full extent of the motion trajectory, a limitation inherent to its reduced temporal sampling. In addition, the Cos^6^ breathing pattern, which models human respiration with longer dwell times near exhalation and shorter durations near inhalation, results in higher voxel intensities in the superior (exhalation) region, as seen in Figure 1.

Among the four reconstruction algorithms (FDK, iCBCT, iCBCT Acuros, and iCBCT MAR), no statistically significant differences were observed in ITV volume or HU values. This consistency across reconstruction methods suggests that all algorithms provide comparable geometric and dosimetric accuracy under both large and small‐motion conditions, demonstrating that image quality and motion representation are stable across clinically available reconstruction options.

When contouring the ITV for comparison, some variability in ITV delineation is inevitable, as in other manual contouring studies, particularly for CBCT images with broader HU distributions. A standardized, peer‐reviewed workflow was implemented to ensure consistency, though minor differences are unavoidable. In addition, a statistical comparison was conducted to confirm significant HU distinctions between the ITV and non‐ITV regions. Also, minor HU variations between the examined scans and the reference scans can be due to the slight difference in x‐ray spectra between HyperSight (125 kVp) and 4DCT (120 kVp).

It is important to recognize that CBCT acquisition relies on sequential angular projections collected over a finite period. When the target is in motion, the reconstructed images are susceptible to distortion due to angular undersampling. Although the motion trajectory can still be visualized at smaller amplitudes, geometric inaccuracies are introduced, as reflected in the Dice coefficient results in Table 1, where values remain around 0.8 even for 5 mm and 10 mm amplitudes. A more pronounced example of this distortion is illustrated in Figure 4c.

In investigating the impact of respiratory motion characteristics on image acquisition, different cycle times, breathing patterns, and scan initiation times were simulated. While it is not possible to model all scenarios, this study provides benchmark data and insights into the influence of motion on CBCT imaging. In Section 3.3, the results for the 6‐ and 8‐second cycle times are comparable. This outcome is expected, as even with an 8 s breathing cycle, the target can traverse from peak to valley within the 6 s acquisition time, allowing the trajectory to be captured when motion amplitude is limited. However, at larger amplitudes, the temporal sampling is insufficient to fully represent the trajectory. Breathing cycles longer than 8 s are uncommon in typical clinical practice and were therefore not evaluated. For different breathing patterns, targets with sinusoidal and hysteresis motion moved more uniformly than those with Cos^6^ motion, allowing more of the trajectory to be visualized at 15 mm amplitude, although the reconstructed images remained distorted. It is important to note that Cos^6^ may more closely approximate typical human breathing. Among the simulated patterns, hysteresis produced the poorest results, as even at 10 mm amplitude, the full motion trajectory could not be captured. In regular motion, each spatial location is typically traversed at least twice, whereas in irregular motion, some locations may not be sampled adequately, leading to volume underestimation, particularly when the cycle duration approaches the acquisition time. Variation in scan initiation time produced different motion representations, as shown in Figure 5, with the degree of blurring and distortion depending on which angular projections coincided with specific motion phases. Overall, ITV size and HU values were consistent across both small and large amplitudes under a 6‐second cycle time. However, for longer respiratory cycles (8 s), the influence of scan initiation became more pronounced, as certain portions of the motion trajectory were not adequately sampled within the short acquisition window. This resulted in visible differences in target position and image appearance across repeated scans.

Clinically, these findings have important implications. While HyperSight CBCT may serve as a suitable planning image for online adaptive workflows involving low‐amplitude, short‐cycle, and regular respiratory motion, it appears less adequate for other scenarios unless supplemented with additional motion management strategies, such as 4D CBCT. Although slow CBCT could introduce reconstruction artifacts, it may still be more appropriate in scenarios with high‐amplitude motion due to its ability to better capture the full extent of target trajectories. These observations are consistent with AAPM Task Group 76 and prior publications,^16^, ^19^, ^20^ which emphasize that motion‐inclusive imaging datasets, such as 4DCT MIP, AIP, slow scans, and respiratory‐binned reconstructions, are essential for robust ITV delineation and reliable dose calculation. MIP images are commonly used to capture the full extent of motion for ITV definition, whereas AIP datasets provide motion‐averaged information more suitable for dose calculation. Similarly, respiratory‐binned reconstructions and slow scans can better represent motion trajectories at the expense of potential image noise or artifacts. In this context, our results indicate that HyperSight rapid scans are sufficient for ITV definition and dose calculation in cases of small respiratory amplitude with regular breathing patterns and short cycle times, whereas ITV underestimation and HU inconsistencies at larger or irregular motion may lead to clinically significant dosimetric compromise if not appropriately addressed.

It is important to note that the HU analysis in this study was based on a lung‐equivalent phantom, where the target exhibits substantially higher HU values than the surrounding low‐density medium. This setup does not fully replicate clinical scenarios in which tumors are embedded within more complex and heterogeneous anatomies, such as soft‐tissue regions of the abdomen or lungs containing ribs, vessels, and bronchial structures. The simplified phantom configuration may therefore underestimate the potential influence of heterogeneous tissue interfaces on image quality and motion depiction. In addition, the simulated respiratory waveforms represent an idealized breathing pattern with fixed amplitude and cycle time, whereas patient‐specific respiratory traces often vary irregularly between and within cycles. Such irregularities could result in additional blurring or incomplete trajectory reconstruction, potentially further reducing ITV accuracy. The present results provide a foundation for future investigations using other anthropomorphic 4D phantoms and clinical datasets to further characterize HyperSight's performance under more complex and realistic motion conditions.

From the results of this study, higher amplitude motion, occurring over a fixed cycle duration, results in faster target movement. Based on the Cos^6^ waveform and a cycle time of 6 s, the average target velocities were calculated as 4.8, 9.2, 13.2, 17.6, and 22.0 mm/s for motion amplitudes of 5, 10, 15, 20, and 25 mm, respectively. Increased target speed reduces temporal sampling at each position, leading to lower voxel intensities, incomplete reconstruction of the motion envelope, and greater image distortion. In clinical scenarios, a more uniform or shorter respiratory cycle may enhance CBCT's ability to detect the full motion trajectory, whereas irregular or prolonged cycles may hinder complete trajectory visualization, potentially leading to an underestimation of the internal target volume and an incomplete representation of the motion trajectory.^5^ This study serves as a foundation for future investigations into the performance of HyperSight CBCT across various clinical scenarios. It also highlights the importance of establishing robust quality assurance protocols for imaging moving targets.

In summary, this study comprehensively evaluates HyperSight CBCT for imaging moving targets, demonstrating that its feasibility for online adaptive radiotherapy depends on motion amplitude, breathing cycle time, and breathing pattern, while revealing limitations at higher amplitudes, longer cycle times, and in less regular motion scenarios. These findings highlight the need for careful clinical implementation and suggest that respiratory motion characteristics should be key considerations when selecting imaging modalities for target delineation and dose calculation in online adaptive radiotherapy.

## 5 CONCLUSION

This study shows that HyperSight CBCT rapid acquisition leads to ITV underestimation and distortion at moderate to large motion amplitudes. Longer cycle times and irregular breathing patterns further compromise trajectory visualization. These findings emphasize that motion amplitude, cycle duration, and breathing pattern should be considered when applying HyperSight CBCT in clinical practice, as inappropriate use in high‐motion scenarios could lead to ITV underestimation and potential dosimetric compromise. Careful patient selection and integration with motion management strategies will therefore be essential for safe and effective clinical implementation, and in cases of large or irregular motion, slow‐scan CBCT may provide a more reliable alternative.

## AUTHOR CONTRIBUTIONS

*Conceptualization and methodology*: Yi‐Fang Wang, Fan Liu, Michael J. Prive, Adam C. Riegel. *Investigation*: Yi‐Fang Wang, Fan Liu. *Formal analysis*: Yi‐Fang Wang, Fan Liu. *Supervision*: Michael J. Prive, Adam C. Riegel. *Writing*: Yi‐Fang Wang, Fan Liu, Michael J. Prive, Adam C. Riegel

## ETHIC STATEMENT

This study did not involve human subjects or identifiable human data; therefore, Institutional Review Board review was not required.

## CONFLICT OF INTEREST STATEMENT

The authors declare no conflicts of interest.

## Supporting information



SUPPORTING INFORMATION

## Data Availability

Research data are stored in an institutional repository and will be shared upon request to the corresponding author.
