# Supplementary material for: Impact of tumor motion on target delineation and dose calculation accuracy using rapid‐acquisition HyperSight CBCT in online adaptive radiotherapy
Source: J Appl Clin Med Phys. 2025 Dec 28;27(1):e70428. doi: 10.1002/acm2.70428 (PMC12744922; doi:10.1002/acm2.70428)
Supplement: Supplementary file 1 — SUPPORTING INFORMATION [file ACM2-27-e70428-s001.pdf]

Appendix A: Representative Breathing Waveforms Used in the Study: Cos^6 (top left), Sinusoidal (middle left), and Hysteresis (bottom left) Patterns, and the Motion Parameters Used in Each Section of the Study (right).

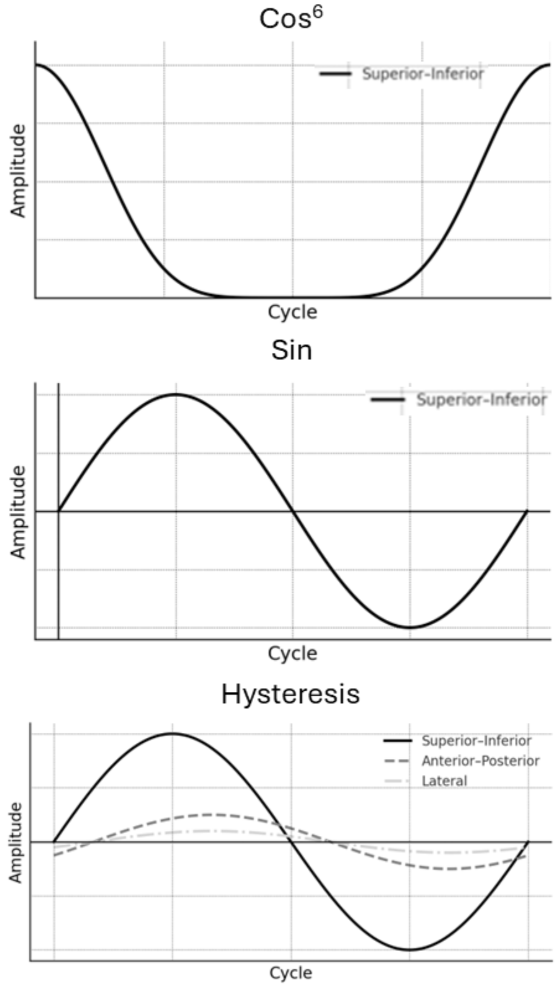

Study Section	Motion Pattern	Cycle Time (s)	Amplitude (mm, SI)	HyperSight CBCT Reconstruction Algorithm(s)
**3.1 ITV and Geometric Comparison**	Cos^6	6	5, 10, 15, 20, 25	FDK, iCBCT, iCBCT Acuros, MAR
**3.2 Image Intensity Evaluation**	Cos^6	6	5, 10, 15, 20, 25	FDK, iCBCT, iCBCT Acuros, MAR
**3.3 Effect of Breathing Cycle Time**	Cos^6	4, 6, 8	5, 10, 15	iCBCT Acuros
**3.4 Effect of Breathing Pattern**	Cos^6, Sinusoidal, Hysteresis	6, 8	5, 10, 15	iCBCT Acuros
**3.5 Effect of Scan Start Timing**	Cos^6 (6 s and 8 s), Sinusoidal (8 s)	6, 8	10, 20	iCBCT Acuros
**3.6 Slow Scan Comparison**	Cos^6	~60	5, 10, 20	iCBCT Acuros
